# A Randomized Trial of Physical Activity in Children and Adolescents with Cancer

**DOI:** 10.3390/cancers13010121

**Published:** 2021-01-02

**Authors:** Paul Saultier, Clothilde Vallet, Frédéric Sotteau, Zeinab Hamidou, Jean-Claude Gentet, Vincent Barlogis, Catherine Curtillet, Arnauld Verschuur, Gabriel Revon-Riviere, Claire Galambrun, Hervé Chambost, Pascal Auquier, Gérard Michel, Nicolas André

**Affiliations:** 1 Department of Pediatric Hematology, Immunology and Oncology, La Timone Children’s Hospital, APHM, 13385 Marseille, France; jean-claude.gentet@ap-hm.fr (J.-C.G.); vincent.barlogis@ap-hm.fr (V.B.); catherine.curtillet@ap-hm.fr (C.C.); arnauld.verschuur@ap-hm.fr (A.V.); gabriel.revon-riviere@ap-hm.fr (G.R.-R.); claire.galambrun@ap-hm.fr (C.G.); herve.chambost@ap-hm.fr (H.C.); gerard.michel@ap-hm.fr (G.M.); nicolas.andre@ap-hm.fr (N.A.); 2 C2VN, Aix Marseille Univ, INSERM, INRAe, 13385 Marseille, France; 3 Association ‘Sourire à la Vie’, 13016 Marseille, France; valletclothilde@gmail.com (C.V.); frederic@sourirealavie.fr (F.S.); 4 CERESS, Aix Marseille Univ, 13385 Marseille, France; zeinab.hamidou@univ-amu.fr (Z.H.); pascal.auquier@univ-amu.fr (P.A.); 5 Department of Public Health, La Timone Hospital, APHM, 13385 Marseille, France; 6 CRCM, Institut Paoli-Calmettes, Aix Marseille Univ, CNRS, INSERM, 13009 Marseille, France

**Keywords:** child, neoplasms, sports, exercise tolerance, quality of life, randomized controlled trials

## Abstract

**Simple Summary:**

Malignant diseases and anticancer treatments alter physical activity and performance in children and adolescents. Physical inactivity may cause both early and long-term complications, increasing the disease-associated burden. However, data on the safety and efficacy of physical activity programs in children with cancer are still scarce. In this randomized controlled open-label clinical trial that included 80 children and adolescents with cancer, the six-minute walk test distance (evaluating exercise capacity) was improved in the intervention group (physical activity program) vs. the control group (86 ± 12 m vs. 32 ± 6 m), a significant difference. Several other physical parameters (flexibility, balance, upper and lower limb strength, trunk and abdominal muscle endurance), global self-esteem and parent-reported quality of life were also better in the intervention group. In children and adolescents undergoing treatment for cancer, a physical activity program is safe, improves exercise capacity, and may have persistent physical and psychological benefits.

**Abstract:**

Background: to evaluate the safety and efficacy of a physical activity program (PAP) in children and adolescents with cancer. Methods: children and adolescents with cancer were randomly assigned in a 1:1 ratio to the six-month PAP (intervention group) or to the control group. The first evaluation was performed at the end of the PAP (T0 + 6 mo). At T0 + 6 mo, both groups received the six-month PAP with a second evaluation at T0 + 12 mo. The primary outcome was the evolution of exercise capacity measured using the six-minute walk test (6 MWT) at T0 + 6 mo. Secondary outcomes included PAP safety and changes in other physical functions, self-esteem, and quality-of-life parameters. Results: The trial involved 80 children and adolescents (age range 5.0–18.4 years), of whom 41 were assigned to the interventional group and 39 to the control group. Underlying malignancies were leukemia (39%) and a broad range of solid tumors (61%). No adverse events occurred. At T0 + 6 mo, the evolution of the 6 MWT distance (±SEM) was improved in the intervention group vs. the control group (86 ± 12 m vs. 32 ± 6 m, *p* < 0.001). Several other physical parameters were significantly improved in the intervention group. Global self-esteem and parent-reported quality-of-life were significantly increased in the intervention group. Analysis at T0 + 12 mo showed persistence of the benefits in the intervention group on exercise capacity evolution (115 ± 18 m vs. 49 ± 11 m, *p* = 0.004) and on most physical and QoL parameters. Conclusion: In children and adolescents with cancer, a physical activity program is safe, improves exercise capacity, and may have physical and psychological benefits.

## 1. Introduction

Malignant diseases and anticancer treatments alter physical activity and performance in children and adolescents [1,2]. Physical inactivity may cause both early and long-term complications, increasing the disease-associated burden [3,4]. In adults, physical activity during or after cancer treatment has been shown to improve physical and psychological parameters [5,6].

Data are limited and discordant regarding the efficacy of physical activity programs (PAPs) during childhood cancer treatments [7]. Some of the previously reported studies are before–after studies with limited sample size [8,9]. Larger prospective cohort studies including 33 to 169 patients suggest interesting benefits of PAPs in terms of physical or quality-of-life (QoL) parameters [10,11]. However, in these studies, the absence of randomization is associated with a high risk of selection bias. Four previously published randomized clinical trials evaluated PAPs in the pediatric cancer population [12,13,14,15]. However, most of these studies were restricted to subgroups of patients, such as those with acute lymphoblastic leukemia [12], solid tumors [13], or patients treated without hematopoietic stem cell transplantation [14]. Speyer et al. suggested that a PAP during hospitalization had a positive impact on health-related QoL [15]. However, this study had a limited sample size (*n* = 30), a short-term endpoint, and did not evaluate physical fitness parameters. Elsewhere, Braam et al. evaluated a combined physical and psychosocial training program in an outpatient setting [14]. This study showed a long-term increase in lower body muscle strength. However, no significant differences were found in terms of aerobic fitness, upper body muscle strength, physical activity, fatigue, or QoL. Due to a relatively low participation rate, it may have been underpowered to detect these between-group differences. Fiuza-Luces et al. published a randomized trial of an early in-hospital PAP including 49 children with malignant solid tumors [13]. These authors showed increased muscle strength but no effect on aerobic capacity, functional capacity, or QoL. Overall, due to small sample size or insufficient study designs, the effects of PAPs for children with cancer remain unclear.

We conducted a randomized controlled open-label trial to evaluate the safety and efficacy of a PAP in children and adolescents treated for cancer.

## 2. Results

### 2.1. Patients Recruitment and Baseline Characteristics

**Figure 1:**
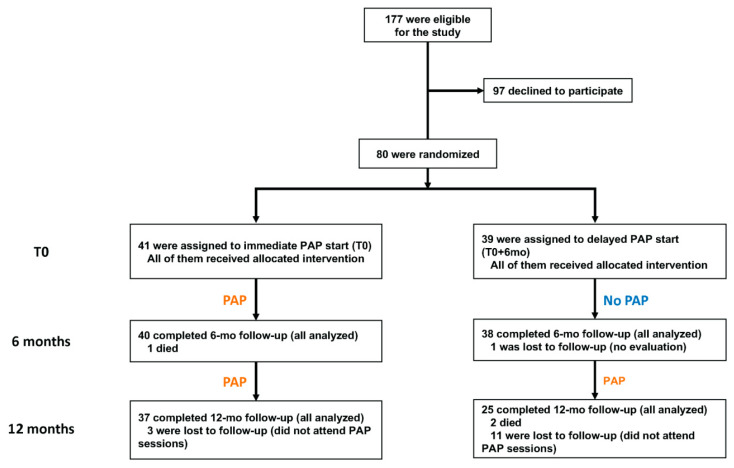
Eligibility, randomization, and follow-up. PAP: physical activity program.

Between November 2014 and January 2018, 177 children and adolescents with cancer were eligible for the study (Figure 1), of which 97 declined to participate. There were no significant differences between the included and eligible but non-included patients (Supplemental Table S1 and Supplemental Figure S1). The trial included 80 cases of mean age 10.4 ± 0.5 years (range 5.0–18.6 years of age). A total of 41 were randomly assigned to the intervention group and 39 to the control group. Baseline characteristics were similar in the two groups (Table 1). The time from primary disease diagnosis to baseline was 10.7 ± 1.2 months. Three patients died during the follow-up due to cancer progression. One and 14 patients were lost to follow-up at T0 + 6 mo and T0 + 12 mo, respectively. Accordingly, data from 78 and 62 cases were available for analysis at T0 + 6 mo and T0 + 12 mo, respectively. At T0 + 12 mo, cases from the control group were significantly more likely to be lost to follow-up compared to the intervention group (*p* = 0.01). Patients lost to follow-up did not attend the PAP sessions.

### 2.2. Physical Endpoints

#### 2.2.1. Change from Baseline to T0 + 6 mo

**Figure 2:**
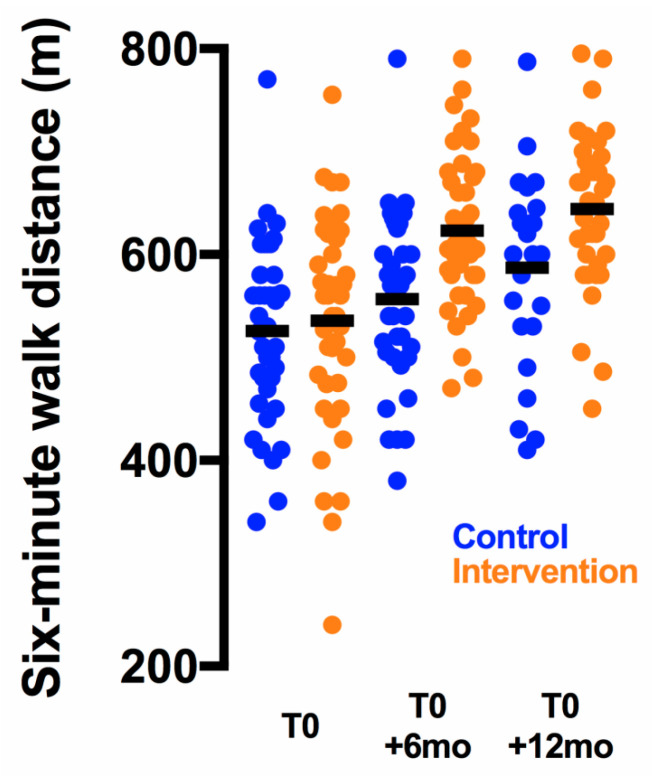
Evolution of the six-minute walk distance. Individual data points are shown; the horizontal lines indicate mean values.

The study showed a greater improvement of exercise capacity from baseline to T0 + 6 mo in the intervention group compared to the control group (six-minute walk test (6 MWT) change 83 ± 12 m vs. 32 ± 6 m, *p* < 0.001) with a very large effect size (ES) of 1.3 (Figure 2 and Table 2). This parameter was the primary endpoint and was available in 78/80 patients (Figure 1). The change in several other physical parameters from baseline was significantly better in the intervention group: flexibility (2.6 ± 0.7 cm vs. 0.7 ± 0.3 cm, *p* = 0.02, ES = 1.0), balance (−2.0 ± 0.5 vs. −0.8 ± 0.2, *p* = 0.048, ES = −0.9), upper limb strength (0.3 ± 0.0 m vs. 0.1 ± 0.0 m, *p* = <0.001, ES = 1.9), lower limb strength (2.4 ± 0.5 cm vs. 0.6 ± 0.3 cm, *p* = 0.04, ES = 1.0 and 24.3 ± 4.4 s vs. 6.0 ± 2.0 s, *p* < 0.001, ES = 1.6 for the Myotest^®^ and chair test, respectively), trunk endurance (23.9 ± 3.4 s vs. 0.6 ± 6.3 s, *p* = 0.001, ES = 0.6), and abdominal muscle endurance (4.9 ± 0.6 vs. 1.7 ± 0.2, *p* < 0.001, ES = 2.7) (Table 2).

#### 2.2.2. Change from Baseline to T0 + 12 mo

The exercise capacity change remained higher in the intervention group (109 ± 15 m vs. 59 ± 9 m, *p* = 0.007, ES = 1.1) (Table 3). Accordingly, the positive effects of the earlier PAP on the following secondary outcomes were still observed: flexibility (3.3 ± 0.8 cm vs. 1.2 ± 0.5 cm, *p* = 0.02, ES = 0.9), balance (−3.3 ± 0.8 vs. −1.1 ± 0.4, *p* = 0.01, ES = −1.1), upper limb strength (0.5 ± 0.1 m vs. 0.3 ± 0.0 m, *p* = 0.009, ES = 1.0), and abdominal muscle endurance (5.8 ± 0.5 vs. 4.3 ± 0.5, *p* = 0.04, ES = 0.6) (Table 3).

### 2.3. Self-Esteem and Quality-of-Life Endpoints

#### 2.3.1. Change from Baseline to T0 + 6 mo

The change in global self-esteem was significantly increased in the intervention group (0.5 ± 0.2 vs. 0.1 ± 0.1, *p* = 0.04, ES = 0.6) (Table 4). The summary score of the parent-reported QoL change was better in the intervention group compared to the control group (6 ± 1 vs. 3 ± 1, *p* = 0.04, ES = 0.8) (Table 4). The leisure activity dimension change was significantly better in the intervention group (18 ± 3 vs. 6 ± 2, *p* = 0.003, ES = 0.9).

#### 2.3.2. Change from Baseline to T0 + 12 mo

Self-esteem change was similar in both trial groups (Table 5). However, the change in the summary score and the three dimensions of the parent-reported QoL were significantly increased in the intervention group compared to the control group: summary score (12 ± 1 vs. 5 ± 2, 0.01, ES = 0.8), vitality (14 ± 2 vs. 8 ± 3, *p* = 0.04, ES = 0.5), leisure activities (30 ± 3 vs. 19 ± 4, *p* = 0.03, ES = 0.6), and physical well-being (18 ± 3 vs. 7 ± 3, *p* = 0.009, ES = 0.8) (Table 5).

### 2.4. Adverse Events

No adverse event occurred during the study.

## 3. Discussion

Although physical activity and performance are altered in children and adolescents with cancer, data on efficacy of PAPs are limited and discordant [7]. In this randomized controlled trial, a six-month PAP significantly improved exercise capacity. Other physical parameters, self-esteem, and QoL were also improved. Despite both groups receiving the PAP for the second six-month period, the improvement of most physical and QoL parameters remained significantly higher in the group that started the PAP earlier.

Low physical activity correlates with cardiovascular risk factors in children and adolescents from the general population [16]. Among other factors, including total body or chest irradiation and anthracycline exposure, decreased physical activity during and after treatment for childhood cancer [2,17] may partly explain the higher risk for cardiovascular diseases among childhood cancer survivors [18]. The 6 MWT was set as the primary outcome evaluation. The test provides a global examination of all the systems involved during exercise. It is a reliable tool to evaluate exercise capacity in both healthy and ill children and is likely to represent the patterns of their daily activities [19,20]. However, other tests, such as the nine-minute run/walk test, the timed up-and-down stairs test, or the timed up-and-go time test, would have been alternative options. At baseline, the exercise capacity of the included patients was ~80% that observed in healthy cases of comparable age [19,21]. This was consistent with a previous study, showing altered 6 MWT performance in children with cancer compared to healthy peers [22]. After one year of PAP, the intervention group exercise capacity appeared to be close (~95%) to that of healthy individuals, which was not the case for the control group (~85%). A precise comparison with age- and sex-matched healthy subjects would be of interest. 

In the general pediatric population, exercise was already shown to increase self-esteem [23]. Using a validated questionnaire [24], we show that the PAP significantly improved self-esteem in children undergoing treatment for cancer. The PAP also improved parent-reported QoL of the patients. Altogether, the combination of restored exercise capacity and increased self-esteem and QoL may promote a long-term practice of physical activities even after PAP discontinuation. Such programs may thus have a significant impact on children’s way of life and in turn on health during survivorship, which will have to be investigated.

This study showed that most of the benefits of the PAP are still significant six months after the end of the randomized intervention. We cannot determine whether these benefits derive from an earlier start of the PAP during the disease course or from a longer PAP time. It is of note that most of the patients of this study completed the PAP after the end of the anticancer treatment. Trial designs allowing the evaluation of longer-term effects of PAP are scarce. Consistent with our results, a randomized control trial involving 68 children showed that the PAP induced an increase in muscle strength eight months after the end of the PAP [14]. This is in line with another study showing that, in the intervention group, two-month detraining values of muscle strength still tended to be higher compared to baseline [13]. However, both studies showed no long-term effect on QoL. In contrast, secondary outcome analysis from the present data suggest persistent benefits of the earlier PAP start in terms of QoL parameters.

Serious adverse events in PAPs did not occur in the previously published randomized trials or cohort study [11,13,14]. We also report no serious adverse event in our trial. It is important to note that the teams involved in PAP research for children with pediatric malignancies often have expertise in adapted physical activity. Specific training of staff should be provided to ensure a safe implementation of PAPs in pediatric cancer centers.

Several factors limit the inclusion of PAPs in pediatric cancer care. Children younger than five years of age can usually not follow a PAP. Notably, almost half of pediatric cancers occur before five years of age. Geographic factors may also hamper the use of tertiary center-based PAPs, which was, however, not the case in the present study. Furthermore, availability and specific training of adapted physical activity instructors is needed for a large implementation of these PAPs in pediatric cancer centers. Finally, these programs often depend on specific funding. Increased PAP standardization and specific recommendations for physical activity during childhood cancer treatment are required and currently under development. Standardization of pediatric PAPs is also required for conducting multicenter trials that would be powered to evaluate the effect of PAPs on survival, as already reported in several cancer types in adults [25,26]. 

Cases from the control group were more likely to be lost to follow-up compared with intervention group. This suggests that patients who start earlier with the PAP may have greater benefit and be more likely to keep doing physical activity. This loss of follow-up was not observed in the previously published trials [13,14]. However, these trials evaluated shorter PAPs, decreasing the risk for loss of follow-up and lack of compliance and, importantly, were not designed with a control group receiving the PAP after a six-month period of standard care. The increased loss to follow-up may be due to the lack of as meaningful engagement with the study team during the initial six months when compared to the control group or due to decreased motivation for physical activity.

Strengths of this study include the size of the included population. This study is the largest randomized controlled trial of PAP in pediatric oncology. Disease stratification and the individually defined PAP start timing allowed the involvement of patients with a broad range of solid tumors and hematological malignancies despite their variable impact on physical capacity. Limitations include that blinding was not possible due to the nature of the intervention. However, the observer and performance bias were minimized using objective outcomes mainly derived from the standard Eurofit battery test. Instructions and verbal encouragements given to the patient prior and during the evaluations were standardized and pre-defined. External validity of the results of this study will have to be confirmed in a multicenter setting.

## 4. Materials and Methods

### 4.1. Trial Design

We conducted a randomized controlled open-label trial involving children and adolescents undergoing treatment for cancer (ClinicalTrials.gov identifier NCT02284061). The study was conducted in the University Hospital of Marseille and promoted by the Assistance Publique—Hôpitaux de Marseille (APHM). Prior to randomization, the treating physician defined for each patient the baseline (T0) as the earliest time point when the patient was considered able to start the PAP. Accounted parameters included disease characteristics, modalities of treatment, tolerance of treatment, and individual fitness.

### 4.2. Participants

Patients aged 5–19 years were eligible for enrollment in the trial if they were treated for a malignant disease in the Department of Pediatric Hematology, Immunology and Oncology of the University Hospital of Marseille, France. Exclusion criteria included a life expectancy ≤18 months, a severe cognitive or psychological deficiency, a length of stay less than 18 months for families living outside the mainland, non-fluency in French, and prior participation in a PAP. The treating physician recruited the patients after informed written consent was obtained from parents and children.

### 4.3. Randomization

Patients were randomly assigned in a 1:1 ratio to an immediate start of the PAP (at T0, interventional group) or a delayed PAP start (six months later, T0 + 6 mo, control group). The randomization list was established prior to study implementation. Patients were stratified according to gender, age group (two modalities: children 5 to 11 years of age and adolescents 12 to 19 years of age), and disease (three modalities: leukemia treated with hematopoietic stem cell transplantation, brain or bone tumor, and other). Patients were allocated within each stratum using random permuted blocks (block size of 4).

### 4.4. Intervention

The study was approved by an institutional review board (“comité de protection des personnes sud méditerranée,” 2014-A01042-45) and was conducted in accordance with the Declaration of Helsinki principles. Adapted physical activity instructors conducted the PAP. The PAP sessions consisted of both in-hospital and outdoor activities. Over six months, the PAP was composed of 30 physical activity sessions of 30 to 90 min (strength and muscle building, balance and proprioception training) and 15 multi-activity sessions of 90 to 240 min (dance, basketball, badminton, yoga, skiing, swimming, paddling, etc.). The sessions were performed during 20 days of physical preparation (department gym, patient’s room, or outdoors) and three stays, including two weekend stays and one long stay of five consecutive days (outdoor camp or stay in the associated premises). All sessions began with a joint, muscle, and cardiac warm-up period, included breaks, and ended with a cool-down period with stretching and relaxation. Intensity was usually set at an estimated 60–70% of maximum heart rate. Intensity was individually adjusted to age, fitness, disease, and treatment, according to the treating physician recommendations. Patients in the control group were offered recreational activities (board games, storytelling, manual and creative activities, and film evenings) from T0 to T0 + 6 mo. At T0 + 6 mo, patients from the intervention group received the PAP for another 6 months, and patients from the control group started the PAP for 6 months.

### 4.5. Trial Endpoints

Endpoints were assessed at baseline (T0), T0 + 6 mo, and T0 + 12 mo by a unique investigator in the department sports gym. Initially planned evaluations at T0 + 3 mo, T0 + 9 mo, and T0 + 18 mo were not performed due to lack of facilities. The primary endpoint was the measure of exercise capacity using the 6 MWT at T0 + 6 mo, as previously described [19,27]. Reference values for 6 MWT in healthy cases were previously published [19,21]. During 6 MWT test and recovery, patients were equipped with a heart-rate monitor (V800, Polar). Standardized instructions and verbal encouragements were administered to the patient at pre-defined time points [27]. To further evaluate physical fitness, we used standardized physical tests mainly derived from the Eurofit test battery [28]. The following physical parameters were assessed: flexibility (sit-and-reach test) [28], balance (flamingo balance test) [28], upper limb strength (1 kg medicine-ball launch), lower limb strength (Myotest^®^ [29] and chair test) [30], trunk muscle endurance (bridge trunk muscle endurance test), and abdominal muscle endurance (sit-up score) [28]. Anthropometric measurements (weight, body mass index, fat mass, lean mass) were measured using an impedance meter. Self-esteem was evaluated using the “Physical Self-Inventory—Very Short Form” (PSI-VSF; 5 dimensions, with a summary score) [24]. The score range was 0–10, with a higher score indicating better self-esteem. Evaluation of the QoL of the included children and adolescents was performed using the parent-reported version of the “Vécu et Santé Perçue de l’Adolescent et de l’enfant” questionnaire (VSP-A; 10 dimensions, with a summary score) [31,32,33]. The score range was 0–100, with higher scores indicating better QoL.

### 4.6. Assessment of Covariates and Adverse Events

Demographic and medical data were collected from patients’ records. Social and familial data and physical activity practice before diagnosis were collected during the baseline interview. The PAP instructors monitored adverse events. Non-protocol physical activity was assessed by self-report at each time point.

### 4.7. Statistical Analyses and Sample Size Calculation

With 80 participants, the trial had 0.8 power to detect a between-group difference of 50 m in the change of the 6-min walk test at T0 + 6 mo, with a two-sided α < 0.05. Categorical variables were summarized using counts and percentages and continuous variables using means and standard error of mean (SEM). Fischer’s exact and χ^2^ tests were used to compare qualitative variables among the intervention and control groups. Quantitative variables were compared using Student’s *t*-test. For all endpoints, changes from baseline (T0) to T0 + 6 mo and from T0 to T0 + 12 mo were compared according to the trial group. Statistical significance was defined as *p* < 0.05. All tests were two-sided. To determine clinical significance of between-group differences, ES were computed as Cohen’s d and interpreted as previously described [34]. Effect sizes of 0.2–0.49, 0.5–0.79, 0.8–1.19, and >1.2 were considered as small, medium, large, and very large, respectively [34,35]. Analyses were performed using PASW Statistics software (v17.0.2).

## 5. Conclusions

Overall, this study supports the implementation of PAPs for children and adolescents with cancer in order to improve exercise capacity.

## Figures and Tables

**Table 1:** Baseline characteristics according to trial group.

Publisher’s Note: MDPI stays neutral with regard to jurisdictional claims in published maps and institutional affiliations. Characteristics	Intervention *n* = 41	Control *n* = 39
**Age** (years)	11.4 ± 0.6	11.2 ± 0.6
**Age group** ^‡^		
Children	25 (61%)	25 (64%)
Adolescents	16 (39%)	14 (36%)
Sex		
Male	23 (56%)	23 (59%)
Female	18 (44%)	16 (41%)
Family		
Parents in couple	31 (76%)	28 (72%)
Single-parent family	10 (24%)	11 (28%)
Number of siblings ^§^		
1–2	27 (66%)	27 (69%)
>2	14 (34%)	12 (31%)
**Distance from home to the treating center** (km)	64 ± 11	57 ± 7
**Physical activity before diagnosis**		
Regular physical activity	38 (93%)	35 (90%)
Sport in a club	25 (61%)	21 (54%)
**Disease**		
Localized	38 (93%)	35 (90%)
Metastatic	3 (7%)	4 (10%)
Primary disease	34 (83%)	37 (95%)
Relapse	7 (17%)	2 (5%)
Leukemia treated with HSCT	5 (12%)	3 (8%)
Brain or bone tumor	12 (29%)	12 (31%)
Other tumor	24 (59%)	24 (62%)
Leukemia	16 (39%)	15 (38%)
Lymphoma	8 (20%)	8 (21%)
Brain tumor	7 (17%)	5 (13%)
Bone tumor	5 (12%)	7 (18%)
Other solid tumor	5 (12%)	4 (10%)
**Anticancer treatment received**		
Chemotherapy	39 (95%)	38 (97%)
HSCT	6 (15%)	5 (13%)
Radiotherapy	11 (27%)	10 (26%)
Surgery	15 (37%)	14 (36%)
**Treatment intensity at intervention (T0)**		
High intensity	26 (63%)	23 (59%)
Maintenance/Metronomic	8 (20%)	11 (28%)
No anticancer treatment	7 (17%)	5 (13%)
Time from diagnosis to intervention (T0) * (months)	11.8 ± 2.0	9.3 ± 1.2

Data are expressed as means ± SEM or *n* (%). There were no significant differences between the intervention and control groups. ^‡^ Children: 5–12 years of age; adolescents: 13–19 years of age. ^§^ Total number of siblings including the patient. * Diagnosis of the primary disease, even for cases with relapsed disease. Abbreviations: HSCT: hematopoietic stem cell transplantation; PAP: physical activity program.

**Table 2:** Physical endpoints at six months.

	Intervention *n* = 40			Control *n* = 38			Group Effect on Change	
Endpoints	Baseline	T0 + 6 mo	Change from Baseline	Baseline	T0 + 6 mo	Change from Baseline	*p*-Value	Effect Size
**Six-minute walk distance** **(primary endpoint)** (m)	540 ± 16	623 ± 12	83 ± 12	525 ± 14	557 ± 13	32 ± 6	**<0.001**	1.3
**Heart rate** (min^−1^)								
Resting	93 ± 2	89 ± 1	−4 ± 2	94 ± 1	91 ± 1	−3 ± 1	0.4	−0.2
End of effort	174 ± 2	173 ± 2	−1 ± 2	173 ± 2	173 ± 2	0 ± 1	0.7	−0.1
Recovery 1′	135 ± 3	137 ± 2	3 ± 3	140 ± 2	136 ± 2	−4 ± 2	0.09	0.7
Recovery 2′	119 ± 2	119 ± 2	0 ± 2	119 ± 1	121 ± 1	2 ± 1	0.3	−0.3
Recovery 3′	111 ± 2	107 ± 2	−5 ± 2	112 ± 2	108 ± 1	−4 ± 1	0.6	−0.1
**Body mass index** (kg.m^−2^)	18.4 ± 0.6	18.8 ± 0.6	0.4 ± 0.2	16.8 ± 0.4	17.2 ± 0.4	0.4 ± 0.2	1.0	0.0
**Fat mass** (kg)	9.2 ± 1.0	9.6 ± 1.2	0.5 ± 0.3	6.7 ± 0.6	7.3 ± 0.7	0.6 ± 0.2	0.7	−0.1
**Lean mass** (kg)	31.6 ± 2.2	32.7 ± 2.2	1.1 ± 0.3	28.2 ± 1.6	28.6 ± 1.6	0.5 ± 0.3	0.1	0.3
**Flexibility** (sit-and-reach test) (cm)	−8.7 ± 1.4	−6.2 ± 1.3	2.6 ± 0.7	−8.8 ± 1.1	−8.1 ± 1.1	0.7 ± 0.3	**0.02**	1.0
**Balance** (flamingo balance test) (A.U.)	7.8 ± 1.4	5.8 ± 1.0	−2.0 ± 0.5	6.7 ± 1.1	5.9 ± 1.0	−0.8 ± 0.2	**0.048**	−0.9
**Upper limb strength** (medicine-ball launch) (m)	3.0 ± 0.2	3.3 ± 0.2	0.3 ± 0.0	2.8 ± 0.1	2.9 ± 0.1	0.1 ± 0.0	**<0.001**	1.9
**Lower limb strength**								
Myotest^®^ (cm)	15.1 ± 1.1	17.5 ± 1.2	2.4 ± 0.5	13.5 ± 0.7	14.1 ± 0.8	0.6 ± 0.3	**0.04**	1.0
Chair test (sec)	45.4 ± 5.3	69.7 ± 7.0	24.3 ± 4.4	35.1 ± 3.9	41.1 ± 3.5	6.0 ± 2.0	**<0.001**	1.6
**Trunk muscle endurance** (bridge trunk muscle endurance test) (sec)	45.4 ± 4.5	69.3 ± 5.7	23.9 ± 3.4	49.8 ± 8.3	50.3 ± 3.4	0.6 ± 6.3	**0.001**	0.6
**Abdominal muscle endurance ** (sit-up score) (A.U.)	9.7 ± 0.8	14.7 ± 0.7	4.9 ± 0.6	9.8 ± 0.7	11.4 ± 0.6	1.7 ± 0.2	**<0.001**	2.7

Data from the first evaluation six months after baseline evaluation (T0 + 6 mo). Data are expressed as means ± SEM. Significant intergroup differences (*p* < 0.05) are highlighted. Abbreviation: A.U.: arbitrary unit.

**Table 3:** Physical endpoints at 12 months.

	Intervention *n* = 37			Control *n* = 25			Group Effect on Change	
Endpoints	Baseline	T0 + 12 mo	Change from Baseline	Baseline	T0 + 12 mo	Change from Baseline	*p*-Value	Effect Size
**Six-minute walk distance** (m)	535 ± 17	644 ± 13	109 ± 15	528 ± 17	587 ± 19	59 ± 9	0.007	1.1
**Heart rate** (min^−1^)								
Resting	92 ± 2	88 ± 1	−4 ± 2	94 ± 2	91 ± 1	−3 ± 2	0.8	−0.1
End of effort	173 ± 2	173 ± 2	0 ± 3	172 ± 3	175 ± 2	3 ± 2	0.3	−0.3
Recovery 1′	134 ± 4	135 ± 2	2 ± 4	138 ± 3	135 ± 2	−3 ± 2	0.4	0.4
Recovery 2′	118 ± 2	117 ± 2	−1 ± 3	119 ± 1	120 ± 2	1 ± 2	0.6	−0.2
Recovery 3′	111 ± 2	106 ± 2	−5 ± 2	112 ± 2	108 ± 2	−4 ± 3	0.8	−0.1
**Body mass index** (kg.m^−2^)	18.4 ± 0.6	19.1 ± 0.6	0.7 ± 0.2	16.9 ± 0.5	17.9 ± 0.5	1.0 ± 0.3	0.3	−0.2
**Fat mass** (kg)	9.0 ± 1.0	9.7 ± 1.0	0.7 ± 0.4	7.3 ± 0.9	9.0 ± 1.5	1.7 ± 1.0	0.3	−0.2
**Lean mass** (kg)	30.5 ± 2.2	32.9 ± 2.1	2.4 ± 0.3	27.4 ± 2.0	29.6 ± 2.0	2.2 ± 0.4	0.7	0.1
**Flexibility** (sit-and-reach test) (cm)	−9.3 ± 1.5	−6.0 ± 1.4	3.3 ± 0.8	−9.0 ± 1.4	−7.8 ± 1.4	1.2 ± 0.5	0.02	0.9
**Balance** (flamingo balance test) (A.U.)	7.9 ± 1.5	4.7 ± 0.9	−3.3 ± 0.8	5.7 ± 1.2	4.6 ± 1.1	−1.1 ± 0.4	0.01	−1.1
**Upper limb strength** (medicine-ball launch) (m)	3.0 ± 0.2	3.4 ± 0.2	0.5 ± 0.1	2.8 ± 0.1	3.1 ± 0.1	0.3 ± 0.0	0.009	1.0
**Lower limb strength**								
Myotest^®^ (cm)	14.7 ± 1.1	17.8 ± 1.3	3.2 ± 0.6	13.9 ± 0.8	15.5 ± 1.0	1.6 ± 0.6	0.08	0.6
Chair test (sec)	44.0 ± 5.5	73.7 ± 8.7	29.7 ± 7.3	29.9 ± 2.9	52.0 ± 4.7	22.1 ± 4.6	0.5	0.4
**Trunk muscle endurance** (bridge trunk muscle endurance test) (sec)	45.1 ± 4.9	74.3 ± 5.7	29.2 ± 3.6	43.8 ± 4.7	67.6 ± 4.4	23.7 ± 3.3	0.3	0.3
**Abdominal muscle endurance** (sit-up score) (A.U.)	9.5 ± 0.8	15.3 ± 0.7	5.8 ± 0.5	10.0 ± 0.8	14.3 ± 0.7	4.3 ± 0.5	0.04	0.6

Data from the second evaluation 12 months after baseline evaluation (T0 + 12 mo). Data are expressed as means ± SEM. Significant intergroup differences (*p* < 0.05) are highlighted. Abbreviation: A.U.: arbitrary unit.

**Table 4:** Self-esteem and quality-of-life endpoints at six months.

	Intervention *n* = 37			Control *n* = 33			Group Effect on Change	
Endpoints	Baseline	T0 + 6 mo	Change from Baseline	Baseline	T0 + 6 mo	Change from Baseline	*p*-Value	Effect Size
**Self-esteem** (PSI-VSF scale) (A.U.)								
Global self-concept	4.4 ± 0.2	5.0 ± 0.1	0.5 ± 0.2	4.6 ± 0.1	4.8 ± 0.1	0.1 ± 0.1	0.04	0.6
Physical self-worth	4.4 ± 0.2	4.6 ± 0.1	0.2 ± 0.1	4.1 ± 0.2	4.3 ± 0.1	0.2 ± 0.1	1.0	0.0
Physical strength	4.0 ± 0.1	4.2 ± 0.1	0.2 ± 0.1	4.0 ± 0.2	4.0 ± 0.1	0.0 ± 0.1	0.2	0.3
Physical attractiveness	3.9 ± 0.2	4.2 ± 0.2	0.2 ± 0.1	3.8 ± 0.2	4.0 ± 0.2	0.2 ± 0.1	0.8	0.1
Physical condition	3.8 ± 0.2	4.1 ± 0.2	0.3 ± 0.1	3.8 ± 0.2	4.1 ± 0.1	0.3 ± 0.1	0.9	0.0
Sport competence	4.1 ± 0.2	4.5 ± 0.1	0.4 ± 0.1	4.3 ± 0.2	4.3 ± 0.1	0.0 ± 0.1	0.08	0.5
**Parent-reported quality of life** (VSP-A parents scale) (A.U.)								
Relationship with parents	80 ± 2	80 ± 2	0 ± 2	84 ± 2	83 ± 2	0 ± 2	1.0	0.0
Body image	69 ± 4	76 ± 3	7 ± 2	77 ± 3	79 ± 3	2 ± 2	0.2	0.4
Vitality	67 ± 2	74 ± 2	7 ± 2	68 ± 2	71 ± 2	3 ± 2	0.13	0.5
Relationship with friends	63 ± 3	67 ± 3	5 ± 3	59 ± 4	64 ± 3	5 ± 4	0.8	0.0
Leisure activities	37 ± 4	55 ± 3	18 ± 3	41 ± 4	47 ± 3	6 ± 2	0.003	0.9
Psychological well-being	68 ± 3	74 ± 3	6 ± 2	74 ± 3	75 ± 3	2 ± 1	0.17	0.5
Physical well-being	52 ± 3	61 ± 2	9 ± 2	52 ± 3	58 ± 3	6 ± 2	0.4	0.2
School performance	76 ± 3	78 ± 3	2 ± 2	79 ± 3	80 ± 3	2 ± 2	1.0	0.0
Relationship with teachers	84 ± 2	86 ± 2	2 ± 2	86 ± 2	89 ± 2	3 ± 1	0.7	-0.1
Relationship with medical staff	82 ± 2	86 ± 2	3 ± 2	87 ± 3	87 ± 3	0 ± 4	0.6	0.1
Summary score	68 ± 2	74 ± 1	6 ± 1	71 ± 2	73 ± 2	3 ± 1	0.04	0.8

Data from the first evaluation six months after baseline evaluation (T0 + 6 mo). Data are expressed as means ± SEM. Significant intergroup differences (*p* < 0.05) are highlighted. Abbreviation: A.U.: arbitrary unit; PSI-VSF: Physical Self-Inventory—Very Short Form; VSP-A: Vécu et Santé Perçue de l’Adolescent et de l’enfant.

**Table 5:** Self-esteem and quality-of-life endpoints at 12 months.

	Intervention *n* = 33			Control *n* = 23			Group Effect on Change	
Endpoints	Baseline	T0 + 12 mo	Change from Baseline	Baseline	T0 + 12 mo	Change from Baseline	*p*-Value	Effect Size
**Self-esteem** (PSI-VSF scale) (A.U.)								
Global self-concept	4.4 ±0.2	5.1 ± 0.1	0.7 ± 0.2	4.7 ± 0.2	5.1 ± 0.1	0.4 ± 0.2	0.4	0.3
Physical self-worth	4.3 ± 0.2	4.7 ± 0.2	0.4 ± 0.2	4.2 ± 0.2	4.8 ± 0.1	0.5 ± 0.2	0.3	−0.3
Physical strength	4.0 ± 0.2	4.3 ± 0.1	0.3 ± 0.2	4.0 ± 0.2	4.2 ± 0.1	0.2 ± 0.2	0.8	0.1
Physical attractiveness	3.9 ± 0.2	4.3 ± 0.2	0.4 ± 0.2	4.0 ± 0.3	4.3 ± 0.2	0.3 ± 0.3	0.9	0.0
Physical condition	3.8 ± 0.2	4.4 ± 0.2	0.6 ± 0.2	3.8 ± 0.2	4.3 ± 0.1	0.5 ± 0.2	0.6	0.2
Sport competence	4.1 ± 0.2	4.7 ± 0.2	0.6 ± 0.2	4.2 ± 0.2	4.5 ± 0.2	0.3 ± 0.2	0.3	0.3
**Parent-reported quality of life** (VSP-A parents scale) (A.U.)								
Relationship with parents	79 ± 2	83 ± 2	4 ± 2	84 ± 2	83 ± 2	−1 ± 2	0.10	0.5
Body image	71 ± 4	83 ± 3	13 ± 3	79 ± 3	88 ± 2	10 ± 3	0.6	0.2
Vitality	66 ± 2	81 ± 2	14 ± 2	70 ± 3	78 ± 2	8 ± 3	**0.04**	0.5
Relationship with friends	63 ± 3	73 ± 2	10 ± 4	62 ± 4	72 ± 3	9 ± 3	0.6	0.1
Leisure activities	38 ± 4	68 ± 3	30 ± 3	42 ± 5	61 ± 3	19 ± 4	**0.03**	0.6
Psychological well-being	69 ± 3	81 ± 2	13 ± 3	74 ± 3	80 ± 2	6 ± 2	0.12	0.6
Physical well-being	50 ± 3	68 ± 2	18 ± 3	56 ± 4	62 ± 2	7 ± 3	**0.009**	0.8
School performance	77 ± 3	78 ± 3	2 ± 3	81 ± 3	80 ± 3	−1 ± 3	0.6	0.1
Relationship with teachers	84 ± 3	86 ± 3	2 ± 3	84 ± 3	88 ± 3	4 ± 2	0.5	−0.2
Relationship with medical staff	83 ± 3	89 ± 3	5 ± 3	89 ± 4	88 ± 3	−1 ± 4	0.3	0.3
Summary score	68 ± 2	80 ± 1	12 ± 1	73 ± 2	79 ± 2	5 ± 2	**0.01**	0.8

Data from the second evaluation 12 months after baseline evaluation (T0 + 12 mo). Data are expressed as means ± SEM. Significant intergroup differences (*p* < 0.05) are highlighted. Abbreviation: A.U.: arbitrary unit; PSI-VSF: Physical Self-Inventory—Very Short Form; VSP-A: Vécu et Santé Perçue de l’Adolescent et de l’enfant.

## Data Availability

The data presented in this study are available on request from the corresponding author.

## Supplementary Materials

The following are available online at https://www.mdpi.com/2072-6694/13/1/121/s1, Supplemental Table S1: Comparison between included and eligible but non-included patients; Supplemental Figure S1: Places of residence of included and eligible but non-included patients.
