# Supplementary material for: A Randomized Trial of Physical Activity in Children and Adolescents with Cancer
Source: Cancers (Basel). 2021 Jan 2;13(1):121. doi: 10.3390/cancers13010121 (PMC7795208; doi:10.3390/cancers13010121)
Supplement: Supplementary file 1 [file cancers-13-00121-s001.pdf]

Supplementary Materials: A Randomized Trial of Physical Activity in Children and Adolescents with Cancer

Paul Saultier, Clothilde Vallet, Frédéric Sotteau, Zeinab Hamidou, Jean-Claude Gentet, Vincent Barlogis, Catherine Curtillet, Arnauld Verschuur, Gabriel Revon-Riviere, Claire Galambrun, Hervé Chambost, Pascal Auquier, Gérard Michel and Nicolas André

Table S1. Comparison between included and eligible but non-included patients.

Characteristics	Included Patients	Eligible but Non-Included Patients
	n = 80	n = 97
**Age at diagnosis** (years)	10.4 ± 0.5	11.6 ± 0.4
**Sex**		
Male	46 (58%)	54 (56%)
Female	34 (43%)	43 (44%)
**Distance from home to the treating center** (km)	60 ± 7	74 ± 8
**Disease**		
Leukemia treated with HSCT	8 (10%)	8 (8%)
Brain or bone tumor	24 (30%)	24 (25%)
Other tumor	48 (60%)	65 (67%)
Leukemia	31 (39%)	33 (34%)
Lymphoma	16 (20%)	27 (28%)
Brain tumor	12 (15%)	11 (11%)
Bone tumor	12 (15%)	13 (13%)
Other solid tumor	9 (11%)	13 (13%)
**Anticancer treatment**		
Chemotherapy	77 (96%)	93 (96%)
HSCT	11 (14%)	12 (12%)
Radiotherapy	21 (26%)	31 (32%)
Surgery	29 (36%)	27 (28%)

Data are expressed as means ±SEM or n (%). There were no significant differences between the included and eligible but non-included patients. Abbreviations: HSCT: hematopoietic stem cell transplantation.

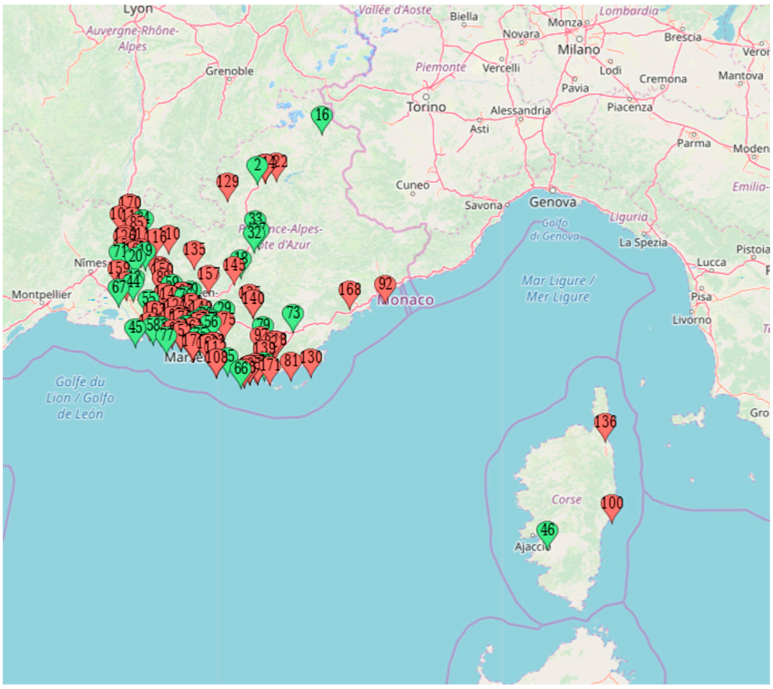

Figure S1. Places of residence of included and eligible but non-included patients. Places of residence of included and eligible but non-included patients are shown in green and red, respectively. Computed with MapCustomizer (https://www.mapcustomizer.com).

© 2021 by the authors. Submitted for possible open access publication under the terms and conditions of the Creative Commons Attribution (CC BY) license (http://creativecommons.org/licenses/by/4.0/).
